# Targeting SARS-CoV-2 Macrodomain-1 to Restore the Innate Immune Response Using In Silico Screening of Medicinal Compounds and Free Energy Calculation Approaches

**DOI:** 10.3390/v15091907

**Published:** 2023-09-12

**Authors:** Anwar Mohammad, Eman Alshawaf, Hossein Arefanian, Sulaiman K. Marafie, Abbas Khan, Dong-Qing Wei, Fahd Al-Mulla, Jehad Abubaker

**Affiliations:** 1 Department of Biochemistry and Molecular Biology, Dasman Diabetes Institute, Dasman 15462, Kuwait; eman.alshawaf@dasmaninstitute.org (E.A.); sulaiman.marafie@dasmaninstitute.org (S.K.M.); jehad.abubakr@dasmaninstitute.org (J.A.); 2 Department of Immunology and Microbiology, Dasman Diabetes Institute, Dasman 15462, Kuwait; hossein.arefanian@dasmaninstitute.org; 3 Department of Bioinformatics and Biological Statistics, School of Life Sciences and Biotechnology, Shanghai Jiao Tong University, Shanghai 200240, China; abbaskhan@sjtu.edu.cn (A.K.); dqwei@sjtu.edu.cn (D.-Q.W.); 4 Department of Genetics and Bioinformatics, Dasman Diabetes Institute, Dasman 15462, Kuwait; fahd.almulla@dasmaninstitute.org; 5 Translational Research Department, Dasman Diabetes Institute, Dasman 15462, Kuwait

**Keywords:** SARS-CoV-2, NSP3, macrodomain-I, medicinal compounds, computational biology

## Abstract

Among the different drug targets of SARS-CoV-2, a multi-domain protein known as NSP3 is a critical element of the translational and replication machinery. The macrodomain-I, in particular, has been reported to have an essential role in the viral attack on the innate immune response. In this study, we explore natural medicinal compounds and identify potential inhibitors to target the SARS-CoV-2–NSP3 macrodomain-I. Computational modeling and simulation tools were utilized to investigate the structural-dynamic properties using triplicates of 100 ns MD simulations. In addition, the MM/GBSA method was used to calculate the total binding free energy of each inhibitor bound to macrodomain-I. Two significant hits were identified: 3,5,7,4′-tetrahydroxyflavanone 3′-(4-hydroxybenzoic acid) and 2-hydroxy-3-O-beta-glucopyranosyl-benzoic acid. The structural-dynamic investigation of both compounds with macrodomain-I revealed stable dynamics and compact behavior. In addition, the total binding free energy for each complex demonstrated a robust binding affinity, of ΔG −61.98 ± 0.9 kcal/mol for Compound A, while for Compound B, the ΔG was −45.125 ± 2.8 kcal/mol, indicating the inhibitory potential of these compounds. In silico bioactivity and dissociation constant (K_D_) determination for both complexes further validated the inhibitory potency of each compound. In conclusion, the aforementioned natural products have the potential to inhibit NSP3, to directly rescue the host immune response. The current study provides the basis for novel drug development against SARS-CoV-2 and its variants.

## 1. Introduction

The severe acute respiratory syndrome coronavirus-2 (SARS-CoV-2) is the causative agent of COVID-19, which the World Health Organization (WHO) declared a global pandemic in March 2020. The SARS-CoV-2 virus has a 29.8 kb positive-sense single-stranded RNA genome with 14 open reading frames (ORFs) encoding 29 proteins that include four structural proteins (Envelope (E), Membrane (M), Nucleocapsid (N), and Spike (S) protein), 16 nonstructural proteins (NSPs), and nine accessory proteins [1,2]. Despite the proofreading capacity during replication, the rapid spread of the SARS-CoV-2 virus has led to a high rate of mutations in the viral proteins. This is evident from the emergence of new variants since the start of the pandemic, such as D614G [3,4,5], Alpha (B.1.1.7) [6,7,8], Beta (B.1.351) [9], Gamma (P.1) [10], Delta (B.1.617.2) [11], Epsilon (B.1.427), Eta (B.1.525), Iota (B.1.526) [12], Kappa (B.1.617.1) [13], Mu (B.1.621), Zeta (P.2), and Omicron (B.1.1.529, BA.1–BA.5 lineages) [14,15,16,17], that have been reported around the world [18]. Furthermore, these SARS-CoV-2 variants and strains have evolved and are more efficient in host cell entry and evasion of the immune system.

Advances in clinical research have led to a better understanding of SARS-CoV-2, facilitating the rapid development of a range of vaccines that present considerable efficacy against severe COVID-19 infection. Nonetheless, the emergence of SARS-CoV-2 variants has compromised the efficacy of the developed vaccines [19]. One effective method to address this issue would involve identifying novel small-molecule drug-like ligands against potent viral protein sites to serve as therapeutic agents for treating and managing SARS-CoV-2 infection. Therefore, drug discovery target studies have investigated several potential drug targets, such as papain-like protease (PL^pro^), S-protein, RNA-dependent RNA polymerase (RdRp), and the nsp10–nsp16 complex [20,21,22], in addition to the main protease (Mpro), which was the principal explored target of SARS-CoV-2 [23,24]. Macrodomain I (Mac-I) of the NSP3 is an attractive therapeutic target for treating SARS-CoV-2 [25,26]. This conserved macrodomain lies within NSP3, the largest membrane-associated cysteine protease encoded by the SARS-CoV-2 genome. Mac-I disrupts the innate immune response to increase viral pathogenesis and virulence, making Mac-I an appealing drug target [25,26,27,28].

NSP3 is a multi-domain protein with three Mac-I domains and two SUD-M-like domains critical in the SARS-CoV-2 translational and replication machinery. Mac-I, also known as X-domain, is highly conserved, with an ADP-ribose (ADPr)-binding site reported in many viruses [29,30], and plays an essential role in the viral attack on the innate immune response. Mac-I functions by hydrolyzing mono-ADP-ribose from target proteins by reversing the activity of ADP-ribosyltransferases [28,31,32,33] to counteract the host anti-viral response of ADP-ribosylation [28]. As a consequence, the role of Mac-I in ADP-ribosylation is associated with the degree of viral pathogenicity [28,34]. Therefore, blocking the ADPr binding to Mac-I would decrease viral pathogenicity, as reported for infectious bronchitis virus (IBV) [29].

Mac-I subverts the host immune response by interfering with the IFN pathway and dysregulating the signal transducer and activator of transcription 1 (STAT1) [35,36], suggesting a contribution to the cytokine storm phenomenon [37,38,39]. Consequently, STAT1 is an in vivo target of SARS-CoV-2 Mac-I, which precisely counteracts its mono-ADP-ribosylation with human PARP14 [39]. Additionally, previous studies have reported that targeting Mac-I attenuates NSP3 activity, leading to a significant reduction in viral pathogenesis and an increase in interferon response and viral neutralization [40].

Considering the importance of Mac-I in SARS-CoV-2 pathogenesis, it is a potential druggable target for new anti-viral compounds. In a previous report, Brosey et al. screened PARGi drugs and reported them as effective candidates against Mac-I in SARS-CoV-2 [40]. Further efforts are needed to discover novel broad-spectrum drugs that could efficiently interact with Mac-I to reduce or block ADPr binding. Drugs with such characteristics would debilitate SARS-CoV-2 virulence and help increase the sensitivity of the innate immune response. Molecular screening and fragment-based drug design studies would help discover fragment binders that function as effective Mac-I inhibitors. In this regard, there is a growing interest in employing computational studies to identify novel small-molecule inhibitors. Studies of the chemical composition of natural products are focused on secondary metabolites, mainly polyphenols that include flavonoids, quercetin, and bibenzyl [41]. Some secondary metabolites exhibit diverse biological activities with potential medicinal use [42]. These metabolites are flavonoids, which are reported as effective antioxidants and potent enzyme inhibitors [43].

This report targets the NSP3–Mac-I domain by screening the MPD3 database with 2295 phytochemicals and the East African Natural Compounds Database (EANCDB), which includes 1875 compounds, to inhibit the Mac-I–ADPr binding site. Furthermore, the conformational stability and dynamic features of Mac-I bound to the selected compounds were tested by subjecting each complex to 100 ns molecular dynamic (MD) simulations and the molecular mechanics–generalized Born surface area (MM/GBSA) to extract free binding energies. This study may provide a basis for in vitro and in vivo experiments for novel drug development, which could inhibit the binding of ADPr to Mac-I and NSP3 activity in SARS-CoV-2 research.

## 2. Materials and Methods

### 2.1. Structures, Sequence Retrieval, and Modeling

The Protein Databank (http://www.rcsb.org/ accessed on 21 December 2022) was used to retrieve the X-ray crystal structure of Mac-I using PDB ID 6W02 [39,44]. The Mac-I structure was prepared and minimized using Chimera and AMBER simulation packages using the FF14SB force field. To screen targets against ligand-specific Mac-I, we utilized compounds from the MPD3 database and the East African Natural Compounds Database (EANCDB) [45,46,47].

### 2.2. Molecular Screening of Medicinal Compound Databases

The MPD3 database with 2295 phytochemicals and 1875 EANCDB compounds were retrieved, prepared, and filtered to meet Lipinski rules before the screening [48,49]. The active site information was based on the available X-ray crystal structure of Mac-I–ADPr (PDB ID: 6W02) for screening. The “structure-based screening module” available online on Mcule (https://mcule.com/dashboard/ accessed on 21 December 2022) was used to screen the MPD3 and EANCDB databases. In addition, Lipinski filtration was employed for the top-scoring compounds, after which the AutoDock Vina algorithm was used for screening purposes, and the top-selected compounds were chosen for the induced-fit docking (IFD) approach to remove false positive results [50].

### 2.3. Molecular Dynamic Simulation (MD)

The top complexes were subjected to molecular simulation using AMBER20 by adding water around each complex (OPC water model). The OPC water model has been developed to accurately reproduce various properties of water, including structure, dynamics, and thermodynamic properties. It has been extensively validated against experimental data, providing reliable results in many applications. Moreover, the OPC model is transferable across various conditions, including different temperatures and pressures. The drugs were extracted from the proteins and parameterized using GAFF. For the whole protein simulation, FF19SB was employed and then subjected to minimization [51,52,53]. For minimization, we applied weak harmonic restraints to the protein backbone atoms (Cα, C, N, and O) while keeping the solvent and ions unrestrained, helping maintain the protein’s secondary structure during minimization. The initial energy minimization used algorithms such as steepest descent or conjugate gradient, which help to relax the system and eliminate close contacts. The following energy minimization step was conducted without restraints on the entire protein and solvent, allowing for further relaxation and optimization of the structure. Each minimization was run for 6000 and 4000 steps, respectively. The heating and equilibration of each complex were performed, followed by the production of 100 ns. A linear heating method was employed, gradually increasing the system temperature over a specified time period to ensure a smooth transition to the desired simulation temperature. Heating was applied to efficiently raise the system temperature, allowing for rapid equilibration while avoiding abrupt changes that could lead to structural distortions. The equilibration process started from 0 K and was gradually raised to the desired simulation temperature of 300 K, ensuring an appropriate starting point and enabling the system to reach the target temperature for subsequent simulations. Positional restraints were applied to specific atoms or groups during the equilibration phase to keep them fixed, ensuring stability while allowing other parts of the system to adapt to the changing temperature and relax into a suitable configuration. The equilibration phase was carried out for 50 nanoseconds, providing sufficient time for the system to relax, reach equilibrium, and establish stable interactions at the desired temperature. The constant pH method was used with a solvent pH set to 7 since we wanted to emulate a simulation at physiological pH. Constant pH MD simulation allows the protonation state of ionizable groups in a protein to change during the simulation according to the local electrostatic environment and the actual pH of the solution [54,55]. The protonation state of amino acid continuously changes, where the pKa values of the ionizable groups can then be obtained from the distributions of the protonation states across the time of the MD simulation. The amino acids with two atoms carrying a proton are aspartate (Asp) and glutamate (Glu). The protonation/deprotonation percentages of Asp and Glu are presented in the Supplementary Table S1.

For each complex, the simulation was run three times to achieve accuracy and confirm the reproducibility of the results. The long-range electrostatic interactions were treated with the particle mesh Ewald algorithm with a 10.0 Å cutoff distance, while the covalent bonds were treated with the SHAKE algorithm [52,56]. Finally, the CPPTRAJ package was used to analyze the trajectories, and PMEMD.cuda was used for running the simulations [57].

### 2.4. Binding Free Energy Calculations

Estimating free energy for the interacting small molecules and the target receptor is the most widely used practice to determine accurate binding strengths. It is employed for diverse macromolecule sets such as protein–ligand, protein–protein, or protein–RNA/DNA to precisely estimate the interacting energy [53,54,55,56]. Thus, to determine the binding free energy, the top two hits were subjected to a molecular simulation MM/GBSA approach employed using the simulation trajectory [58]. Along with the total binding energy (G), van der Waal (vdW), electrostatic energy, generalized Born (GB), and ESURF were estimated.

### 2.5. Determination of Dissociation Constant and Bioactivity for the Top Hits

Quantifying the binding strength by estimating the dissociation constant (KD) using PRODIGY-LIG (PRODIGY for LIGands) and in silico bioactivity prediction against various classes of druggable proteins informs the selection of the final small molecule with Molinspiration cheminformatics [59].

## 3. Results

### 3.1. Macrodomain I Structural Modeling

**Figure 1:**
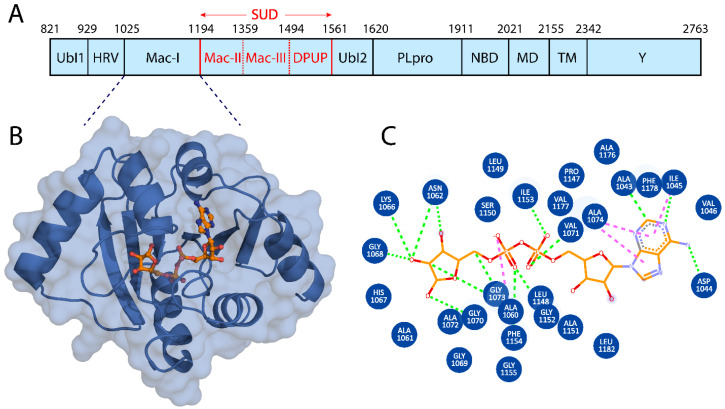
(**A**) Domain structure of NSP3. (**B**) Cartoon representation of Mac-I (blue) with the bound ligand shown in stick form with orange carbons. (**C**) Two-dimensional structure of the drug showing its interaction with Mac-I. Hydrogen bonds are represented by green dashed lines, and pink dashed lines indicate hydrophobic interactions.

The SARS-CoV-2 Nsp3 consists of 1945 amino acids with ten functional domains (Figure 1A), with the Nsp3–Macro domains contributing significantly to inhibit the innate immune response. The Mac-I domain is 169 residues in length, highly conserved [34], and plays an essential role in counteracting host-mediated anti-viral ADPr signaling. The hydrolase activity enables it to remove ADPr from target proteins, and this biochemical feature is directly associated with the SARS-CoV-2 pathogenicity level [34]. The Mac-I domain is an attractive drug target, identifying specific small-molecule inhibitors that would rescue and support the host immune innate response. In our study, we utilized the X-ray crystal structure of the Mac-I domain (PDB ID: 6W02) (Figure 1B) to identify natural compounds from the MPD3 and EANCDB natural product databases that can disrupt the ADPr interactions with Mac-I.

### 3.2. Discovery of Small-Molecule Inhibitors by Screening Large Libraries

The Mac-I-ADPr binding site was targeted with a multi-step computational screening approach using the Mcule [60] and AutoDock Vina docking tools. Initially, for MPD3 and EANCDB, 4170 compounds were retrieved and subjected to ADMET analysis, whereby 2153 compounds obeyed Lipinski’s rule of five. By setting the docking score threshold to ≥−5 kcal/mol, the MPD3 database presented 30 compounds with docking scores ranging between −8.2 and −10.6 kcal/mol, while the EANCDB showed 112 compounds’ docking scores between −6.6 and −10.0 kcal/mol [27]. To further narrow down the selection, the docking threshold was increased to −9.46 kcal/mol, corresponding to the reported score for ADPr docking to Mac-I [27]. The increased threshold resulted in three compounds from MPD3 and eight from the EANCDB database with docking scores higher than −9.46 kcal/mol. The eleven compounds were re-docked with AutoDock Vina with four compounds, resulting in docking scores to Mac-I higher than −9.46 kcal/mol (Table 1). Furthermore, for the top two compounds—3,5,7,4′-tetrahydroxyflavanone 3′-(4-hydroxybenzoic acid) (Compound A) and 2-hydroxy-3-O-beta-glucopyranosyl-benzoic acid (Compound B)—complexes with Mac-I underwent MD simulations to measure their conformational dynamics and stability.

### 3.3. Binding Modes of the Selected Compounds

**Figure 2:**
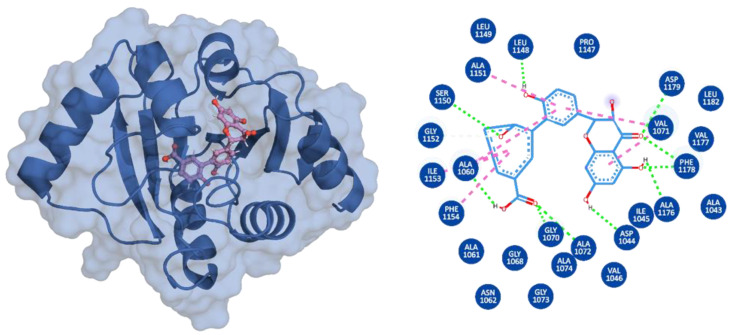
Interaction pattern of 2-hydroxy-3-O-beta-glucopyranosyl-benzoic acid (Compound B). (**A**) Cartoon representation of Mac-I (blue) and the bound ligand shown in stick form with pink carbon atoms. (**B**) Two-dimensional representation of the drug–Mac-I interactions. Hydrogen bonds are represented by green dashed lines; pink dashed lines indicate hydrophobic interactions.

**Figure 3:**
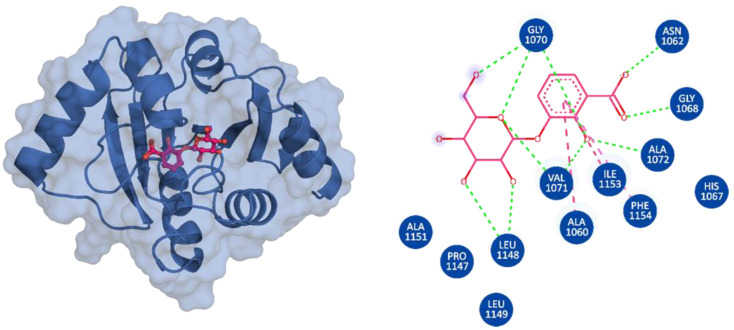
Interaction pattern of 3,5,7,4′-Tetrahydroxyflavanone 3′-(4-hydroxybenzoic acid) (Compound A). (**A**) Cartoon representation of Mac-I (blue) and the bound ligand shown in stick form with purple carbon atoms. (**B**) Two-dimensional representation of the drug–Mac-I interactions. Hydrogen bonds are represented by green dashed lines; pink dashed lines indicate hydrophobic interactions.

3,5,7,4′-Tetrahydroxyflavanone 3′-(4-hydroxybenzoic acid) (Compound A) is an extract from the moss species Hypnum cupressiforme, commonly known as cypress-leaved plait-moss. In general, Bryophyta (mosses) are reported to be rich in active metabolites exhibiting antioxidant, antimicrobial, as well as anti-viral properties (Table 2) [42,61]. Since Compound A is a polyphenolic compound extracted from Hypnum cupressiforme, it is expected to have anti-viral and antimicrobial activities. Compound A, with a docking score of −11.54 kcal/mol, formed three hydrophobic interactions, which included bonds with Ile1153 and two interactions with Phe1154. In addition, 11 hydrogen bonds were formed with residues Gly1068, Gly1070, Val1071, Ala1072, Ser1150, Ala1151, Gly1152, Ile1153, Phe1154, Phe1178, and Asp1179 (Figure 2). 2-hydroxy-3-O-beta-glucopyranosyl-benzoic acid (Compound B) is extracted from the dried stem and roots of the Strychnos cocculoides plant (Table 2). Strychnos cocculoides is widely distributed in tropical regions, and in Tanzanian folk medicine, the root and stem barks are used to treat fevers, stomach pain, and snake bites [62,63]. The roots are also widely used to alleviate eczema and treat infections [64]. The interaction pattern of Compound B with Mac-I resulted in two hydrophobic interactions, including bonds with Ile1153 and Phe1154, while nine hydrogen bonds involving residues Gly1068, Gly1070, Val1071, Ala1072, Leu1148, Ser1150, Ala1151, and Gly1152 (Figure 3) were formed.

### 3.4. Dynamic Stability and Compactness Assessment

**Figure 4:**
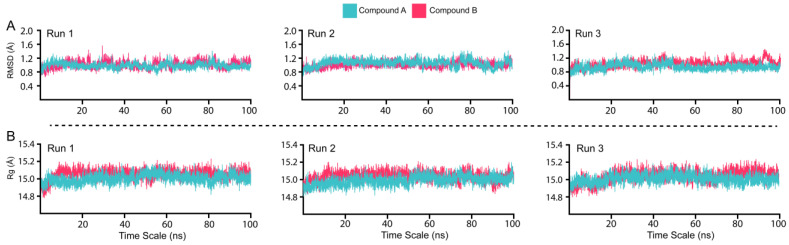
Dynamic stability and compactness assessment of ligands bound to Mac-I. (**A**) RMSD of both the ligands in complex with Mac-I. (**B**) Structural compactness in a dynamic environment.

The conformational stability and dynamic environment of Mac-I bound to Compounds A and B were elucidated by running 100 ns MD simulations of the complexes, with the simulations being run in triplicate to ensure the accuracy of the reproducibility of the results. The root-mean-square deviation (RMSD) trajectories of the Cα-atoms demonstrated each system’s dynamic stability and convergence (Figure 4A). The radius of gyration (Rg) indicates the structural compactness of the Mac-I–ligand complexes as a function of time (Figure 4B). The structural compactness of the interacting partners reveals essential information regarding the binding and unbinding events during the MD simulation.

The complexes of Mac-I with Compounds A and B demonstrated overall stable structures (Figure 4A). In the initial stages of Mac-I in complex with Compound A, the structure converged to 1.2 Å in the first 10 ns. From 10 ns onwards, the complex remained at equilibrium for the duration of the 100 ns simulation. Furthermore, Runs 1, 2, and 3 of Mac-I (Figure 4A turquoise) in complex with Compound A demonstrated similar RMSD atomic configurations. For Mac-I in complex with Compound B (Figure 4A magenta), the RMSD converged to 1.2 Å in the first 15 ns, after which the complex equilibrated and averaged 1.0 Å during the 100 ns simulation. The convergence of the second and third Mac-I–Compound B complex runs showed a similar atomic configuration to the first run, demonstrating the reliability of the MD simulation. For both Mac-I complexes with natural products, the RMSD was maintained with no structural perturbation, revealing a stable binding to the active site residues. Moreover, the average RMSD for both complexes was 1.0 Å, indicating that these ligands form a very stable complex with Mac-I and may inhibit the Mac-I interaction with ADPr, consequently reducing SARS-CoV-2 pathogenesis.

The Rg values of Mac-I binding to Compounds A and B averaged 15.0 Å in both duplicate runs (Figure 4B). This resulted from the stable binding of ligands with minimal unbinding events during the simulation, further corroborated by the RMSD results. The RG data indicate that Compounds A and B may bind Mac-I more favorably than ADPr. The structural compactness strongly aligns with the RMSD results, with no significant variations in the size of the MAC-I complex with Compounds A and B. Consequently, such robust binding indicates the favorable pharmacological properties of both molecules.

### 3.5. Estimation of Hydrogen Bonding and Residual Flexibility

**Figure 5:**
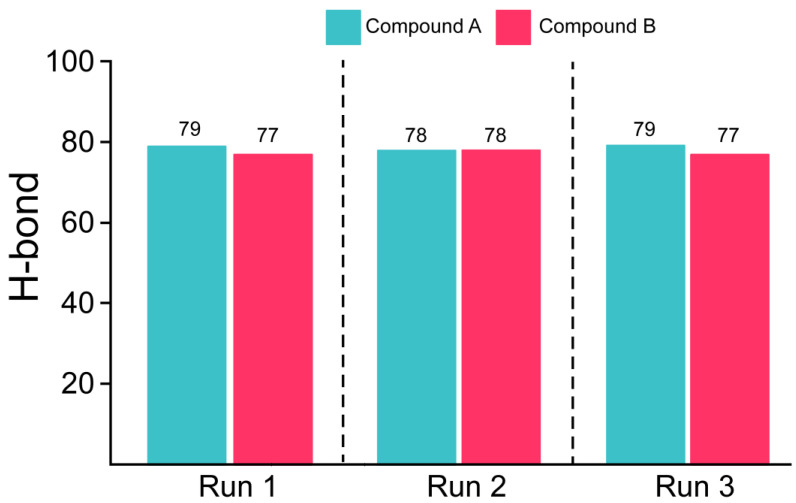
H-bonds of Mac-I bound to Compounds A (magenta) and B (fuchsia). The H-bonds were calculated for each run separately.

Hydrogen bonds (H-bonds) contribute to protein–protein and protein–ligand binding. The determination of H-bonds is vital to ascertaining the intermolecular interactions between proteins and the selected ligands. The number of H-bonds formed between Mac-I and Compounds A and B can estimate the strength of the protein–ligand complex (Figure 5). The average number of H-bonds in the Compound A–Mac-I complex was 79 bonds, while the Compound B–Mac-I complex demonstrated an average of 77 hydrogen bonds. The higher number of hydrogen bonds in the Compound A–Mac-I complex than in the Compound B–Mac-I complex indicated a higher-affinity interaction and potentially more significant inhibitory effect. The H-bond estimation for Runs 2 and 3 revealed a similar bonding network with an average of 75 to 90 bonds for each complex. As such, demonstrating a stable conformation for each system could predict the native binding conformation, too. Overall, the results from each replica validate and reproduce the findings, thus showing the reliability of the results and the anti-viral potential of both compounds.

**Figure 6:**
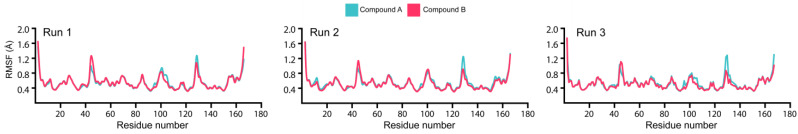
Residue flexibility assessment of Mac-I interacting with Compounds A and B to elucidate the dynamic environment.

The root-mean-square fluctuations (RMSFs) of the Cα-atoms demonstrate the flexibility and average position in a given conformation when the protein is in complex with a protein or ligand (Figure 6). The complexes demonstrated similar residual flexibility, except in regions 41–50, 95–110, and 125–135 of Mac-I, which showed a slight fluctuation. The higher fluctuations may have resulted from the higher conformational sampling in the Mac-I binding pocket. The Mac-I–Compound B complex demonstrated higher fluctuations between residues 40 and 45, with the Compound A–Mac-I complex showing higher fluctuations between residues 95–105 and 125–135. The results for Run 2 and 3 aligned with the results for Run 1, thus showing similar dynamic behavior during simulation. Our findings showed a very low mean RMSF, demonstrating that the residues of Mac-I in complex with Compounds A and B conformed to favorable energy minima [65]. These results are consistent with earlier findings describing low RMSF for the best compounds interacting with SARS-CoV-2 proteins [50].

### 3.6. Binding Free Energy Estimation

The MM/GBSA method is more robust in calculating binding energies than the classical docking scores. In addition, the MM/GBSA approach is computationally affordable compared with the costly alchemical-free energy methods. Previous studies have widely applied this approach to discover potential drug candidates for treating SARS-CoV-2. Henceforth, we employed the MM/GBSA method in the current study to estimate the binding free energy of Compound A and B complexes with Mac-I (Table 3). The MM/GBSA values were calculated for both MD simulation runs, and the average values were compared between Compounds A and B in complex with Mac-I.

Our findings revealed average values of vdW (−68 ± 3.4), electrostatic (−21.1 ± 0.6), EGB (9.0 ± 1.0), ESURF (19.9 ± 3.0), and the total binding energy ΔG (−61.2 ± 1.4) kcal/mol for the Compound A–Mac-I complex. For the Compound B–Mac-I complex, the average values were vdW (−51.2 ± 0.41), electrostatic (−28 ± 6.4), EGB (9.3 ± 4.53), ESUF (25.5 ± 2.31), and total binding energy ΔG (−44.9 ± 2.02) kcal/mol. These findings demonstrate that Compound A, with an average ΔG of −61.98 ± 0.9 kcal/mol, is a more potent natural compound in blocking ADPr binding than Compound B, with an average ΔG of −45.125 ± 2.8 kcal/mol. To further corroborate the above findings, we calculated Mac-I’s entropic values (ΔTS) [65] in complex with Compounds A and B, resulting in a ΔTS of −16.26 and −11.74, respectively. This indicates that Compound A demonstrates a tighter binding with the Mac-I domain, presenting it as a potential drug to bind Mac-I and inhibit NSP3 activity.

### 3.7. In Silico Bioactivity and K_D_ Estimation

**Figure 7:**
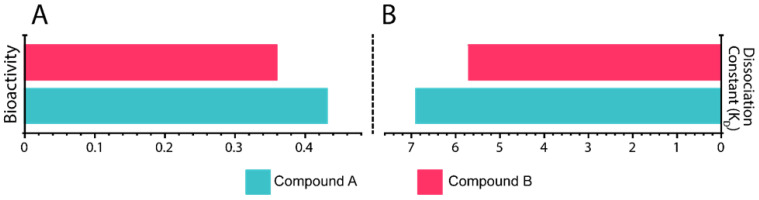
(**A**) In silico bioactivity results for 3,5,7,4′-tetrahydroxyflavanone 3′-(4-hydroxybenzoic acid)–Mac-I and 2-hydroxy-3-O-beta-glucopyranosyl-benzoic acid–Mac-I complexes. (**B**) K_D_ results for 3,5,7,4′ tetrahydroxyflavanone 3′-(4-hydroxybenzoic acid)–Mac-I and 2-hydroxy-3-O-beta-glucopyranosyl-benzoic acid–Mac-I complexes.

The dissociation constant (KD) is the fundamental criterion to elucidate the binding properties of ligands to proteins. In silico PRODIGY-LIG (PRODIGY for LIGands), a post-MD simulation, and an MM/GBSA analysis were used to calculate the KD of Compounds A and B bound to Mac-I (Figure 7A). The Compound A complex with Mac-I showed a KD of –6.9 kcal/mol, whereas Mac-I–Compound B demonstrated a binding affinity of −5.8 kcal/mol. The stronger binding affinity of the latter correlates with the MM/GBSA data calculated from the MD simulations. In addition, in silico bioactivity levels for the complexes were found to be 0.39 and 0.44, respectively (Figure 7A), showing the strong potency of both compounds, with Compound A being the most potent.

## 4. Conclusions

The current study used computational modeling and simulation tools to target the Mac-I domain of SARS-CoV-2. Screening of large libraries such as MPD3 and EANCDB identified two hits: 3,5,7,4′-tetrahydroxyflavanone 3′-(4-hydroxybenzoic acid) (Compound A) and 2-hydroxy-3-O-beta-glucopyranosyl-benzoic acid (Compound B). These drugs can potentially bind to Mac-I and inhibit NSP3 activity, thereby directly rescuing the host immune response. The current study provides a basis for novel drug development against SARS-CoV-2 and its variants.

## Figures and Tables

**Table 1:** Top hits identified through muti-step screening and rescoring via the IFD method. The table presents the 2D structures, compound names, and docking scores of the top four.

2D Structure	Compound Name	IFD Score	Identifier
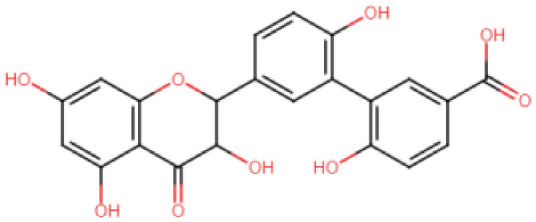	3,5,7,4′-Tetrahydroxy flavanone 3′-(4-hydroxybenzoic acid)	−11.54	A
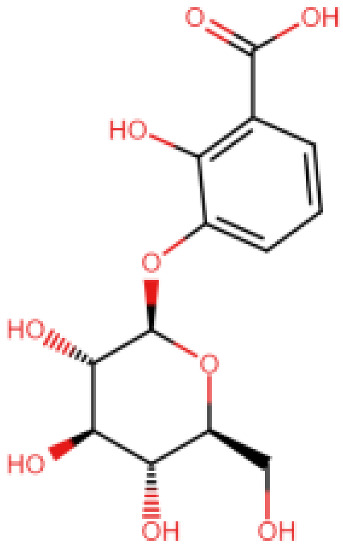	2-Hydroxy-3-O-beta-glucopyranosyl-benzoic acid	−10.0	B
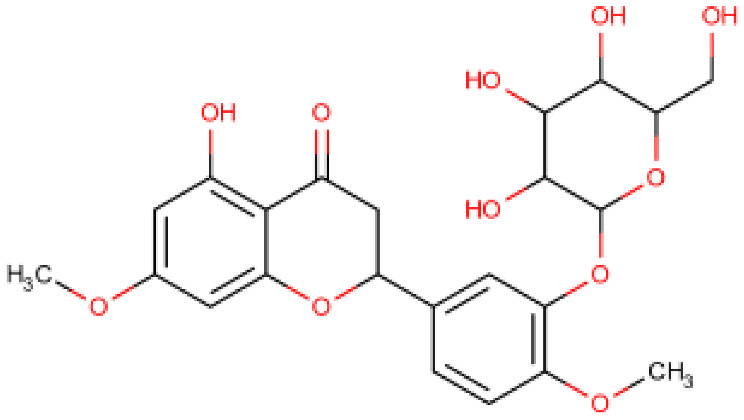	5,3′-Dihydroxy-7,4′-dimethoxyflavanone 3′-glucoside	−9.76	C
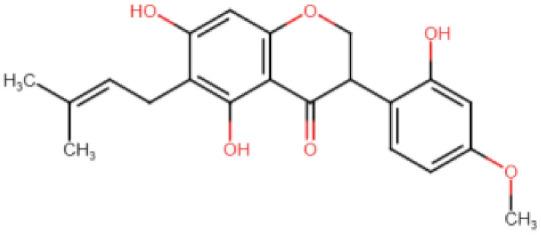	5,7,3′-Trihydroxy-2′,4′-dimethoxy-6-prenylisoflavanone	−9.66	D

**Table 2:** ADMET analysis results and biological and toxicological properties.

Compound	Identifier	MW. (g/mol)	Source	Molecule Class	Biological Activity	Lipinski Violation	AMES * Toxicity	Rat Oral LD50 (mol/kg)	Max. Tolerated Dose (Human) (log Mg/kg/day)	*T. pyriformis* Toxicity ** (log µg/L)	HBD	HBA	Rotatable Bonds No.	TPSA	Bioactivity
3,5,7,4′-Tetrahydroxy flavanone 3′-(4-hydroxybenzoic acid)	A	424.36	*Hypnum cupressiforme*	Flavonoid	Anti-viral	1	-	-	-	-	6	9	3	164.74 Å^2^	0.24
2-hydroxy-3-O-beta-glucopyranosyl-benzoic acid	B	316.26	*Strychnos cocculoides*	Phenolic	Anti-inflammatory	1	No	2.305	1.294	0.285	6	9	4	156.91 Å^2^	0.43

ADMET (Absorption, Distribution, Metabolism, Excretion, and Toxicity). * AMES toxicity test, in-vitro testing to assess the potential carcinogenic effect of chemicals. ** Tetrahymena pyriformis, the most commonly ciliated model, used for toxicological studies.

**Table 3:** Binding free energy calculated as MM/GBSA of Compounds A and B with SD. All the values are presented in kcal/mol.

MM/GBSA	Compound A-Run 1	Compound A-Run 2	Compound A-Run 3	Average	SD	Compound B-Run 1	Compound B-Run 2	Compound B-Run 3	Average	SD
vdW	−71.16	−68.44	−64.37	−68.0	3.4	−50.96	−50.96	−51.67	−51.2	0.41
Electrostatic	−22.77	−21.72	−21.89	−22.1	0.6	−34.13	−28.76	−21.22	−28.0	6.49
ESURF	23.29	18.78	17.64	19.9	3.0	28.10	24.52	23.78	25.5	2.31
EGB	8.05	10.01	8.85	9.0	1.0	13.83	9.29	4.78	9.3	4.53
ΔG Bind	−62.59	−61.37	−59.77	−61.2	1.4	−43.16	−47.09	−44.33	−44.9	2.02

## Data Availability

Data availability upon request.

## Supplementary Materials

The following supporting information can be downloaded at: https://www.mdpi.com/article/10.3390/v15091907/s1, Table S1: Time-dependent distribution of protonation and deprotonation states of Aspartate and Glutamate residues.
