# Supplementary material for: Targeting SARS-CoV-2 Macrodomain-1 to Restore the Innate Immune Response Using In Silico Screening of Medicinal Compounds and Free Energy Calculation Approaches
Source: Viruses. 2023 Sep 12;15(9):1907. doi: 10.3390/v15091907 (PMC10538035; doi:10.3390/v15091907)
Supplement: Supplementary file 1 [file viruses-15-01907-s001.zip › viruses-2536335-supplementary.pdf]

Table S1: Time-dependent distribution of protonation and deprotonation states of Aspartate and Glutamate residues.

Time Interval (ns)	Asp Protonated (%)	Asp Deprotonated (%)	Glu Protonated (%)	Glu Deprotonated (%)
**1-10**	70	30	80	20
**10-20**	65	35	75	25
**20-30**	60	40	70	30
**30-40**	55	45	65	35
**40-50**	50	50	60	40
**50-60**	45	55	55	45
**60-70**	40	60	50	50
**70-80**	35	65	45	55
**80-90**	30	70	40	60
**90-100**	75	25	65	35
